# Field Evaluation of the ClaID PCR System Reveals Predominance of Clade I-Associated Molecular Profiles Among Clinical *Candida auris* Isolates Recovered in İstanbul, Türkiye

**DOI:** 10.3390/antibiotics15070692

**Published:** 2026-07-16

**Authors:** Feray Ferda Şenol, Zülal Aşçı Toraman, Uğur Vural, Zeynep Çelik, Yüksel Akkaya, Mustafa Çilkız, Begüm Nalça Erdin, Sevda Kırbağ, İbrahim Halil Kılıç

**Affiliations:** 1 Department of Medical Microbiology, Elazığ Fethi Sekin City Hospital, Elazığ 23280, Türkiye; drferdasenol@yahoo.com; 2 Department of Medical Microbiology, Faculty of Medicine, Fırat University, Elazığ 23119, Türkiye; zulalasci@firat.edu.tr; 3 Department of Biology, Faculty of Science and Letters, Gaziantep University, Gaziantep 27310, Türkiye; uv211002@mail2.gantep.edu.tr (U.V.); zc211002@mail2.gantep.edu.tr (Z.Ç.); 4 Department of Medical Microbiology, Hamidiye Faculty of Medicine, University of Health Sciences, İstanbul 34668, Türkiye; yuksel.akkaya@sbu.edu.tr; 5 Ankara Poultry Research Institute, Ministry of Agriculture and Forestry, Ankara 06170, Türkiye; mustafa.cilkiz@tarimorman.gov.tr; 6 Department of Medical Microbiology, Darıca State Hospital, Kocaeli 41700, Türkiye; begum.nalcaerdin@saglik.gov.tr; 7 Department of Biology, Faculty of Science, Fırat University, Elazığ 23119, Türkiye; skirbag@firat.edu.tr

**Keywords:** *Candida auris*, molecular epidemiology, clade identification, ClaID, PCR, Türkiye, fungal pathogen

## Abstract

**Background:** *Candida auris* has emerged globally as a multidrug-resistant fungal pathogen responsible for healthcare-associated outbreaks and invasive infections. Whole-genome sequencing studies have demonstrated the existence of genetically distinct clades that differ in geographical distribution, antifungal resistance patterns, virulence traits, and outbreak potential. **Objectives:** This study aimed to evaluate the performance of the ClaID clade identification PCR system among clinical *Candida auris* isolates collected in İstanbul, Türkiye, and to investigate the clade-associated molecular profiles of circulating isolates. **Methods:** Forty-four clinical *C. auris* isolates were analysed using the auris universal sequence (AUS) assay and clade-specific sequence assays (CSS1–CSS5). PCR amplification results were interpreted according to the ClaID framework. **Results:** AUS amplification was detected in 41/44 isolates (93.2%). CSS1 amplification was observed in 39/44 isolates (88.6%), indicating a predominance of Clade I-associated molecular profiles within this regional İstanbul isolate collection. No amplification was detected using CSS2, CSS3, CSS4, or CSS5 assays. Three isolates were AUS-negative and five isolates did not yield CSS1 amplification despite repeated testing. **Conclusions:** The findings suggest that the majority of analyzed clinical isolates from İstanbul exhibited Clade I-associated molecular profiles rather than definitive WGS-confirmed clade assignments. This study provides one of the first field evaluations of the ClaID system in a Turkish clinical isolate collection and contributes regional molecular epidemiological data regarding PCR-based clade-associated profiles of *C. auris* in Türkiye.

## 1. Introduction

*Candida auris* is an emerging multidrug-resistant fungal pathogen that has become a major global public health concern since its first description in 2009 from Japan [1]. During the past decade, *C. auris* has been reported from more than 50 countries across six continents and has been associated with numerous healthcare-associated outbreaks, prolonged hospital transmission, and invasive infections with high mortality rates [2,3,4,5] Unlike many other Candida species, *C. auris* exhibits remarkable environmental persistence, efficient patient-to-patient transmission, and frequent resistance to multiple antifungal drug classes, making infection prevention and control particularly challenging [6,7,8].

Whole-genome sequencing studies have revealed substantial genetic diversity within *C. auris* and demonstrated the existence of five major phylogeographic clades, designated Clades I–V [2,9,10]. These clades are separated by thousands of single nucleotide polymorphisms, indicating long-term evolutionary divergence despite the relatively recent global emergence of the species [2,11]. Clade I (South Asian), Clade II (East Asian), Clade III (African), Clade IV (South American), and Clade V (Iranian) have distinct geographical origins and exhibit differences in antifungal susceptibility profiles, virulence-associated characteristics, biofilm formation capacity, aggregation phenotypes, environmental persistence, and outbreak potential [9,10,12].

The identification of *C. auris* clades has become increasingly important for molecular epidemiology, outbreak investigations, and surveillance programmes. Previous studies have demonstrated that Clades I, III, and IV are frequently associated with large hospital outbreaks and multidrug-resistant phenotypes, whereas Clade II isolates are commonly linked to ear infections and appear less frequently involved in healthcare-associated transmission events [2,9,12]. Furthermore, clade-specific resistance mutations in ERG11 and FKS1 have been described, suggesting potential clinical relevance of clade assignment in predicting antifungal susceptibility trends [9,11].

Whole-genome sequencing remains the gold standard for clade determination; however, its implementation is often limited by cost, infrastructure requirements, and bioinformatic expertise [2,11]. Alternative molecular approaches including multilocus sequence typing, short tandem repeat analysis, and allele-specific PCR assays have therefore been developed to facilitate rapid clade assignment [13,14,15]. Among these approaches, the allele-specific PCR assay developed by Carolus et al. enables simultaneous species identification and clade determination through amplification of clade-specific genetic markers [12].

More recently, Narayanan et al. developed the ClaID framework, a rapid colony-PCR-based system that employs clade-specific genomic regions identified through comparative whole-genome analyses to distinguish among the major *C. auris* clades [16]. The system was subsequently expanded to include the recently recognised Iranian Clade V through the development of the CSS5 assay [17]. These advances have created practical opportunities for cost-effective clade assignment in routine laboratory settings and epidemiological investigations.

Despite the increasing number of *C. auris* reports worldwide, molecular epidemiological data regarding clade distribution in Türkiye remain limited. Several clinical studies have documented the emergence of *C. auris* in Turkish healthcare institutions; however, information regarding the genetic background and clade composition of circulating isolates is still scarce. Understanding clade distribution is important for surveillance, outbreak preparedness, and comparison with regional and global epidemiological trends [18,19,20,21].

Recent reports from Türkiye have further emphasized that *C. auris* is no longer limited to sporadic case reports but represents an emerging healthcare-associated pathogen requiring coordinated diagnostic, clinical, and infection-control strategies [2,22]. In this context, rapid clade-level molecular screening may complement conventional identification methods by supporting earlier recognition of circulating lineages and strengthening national surveillance efforts [16,17,22].

Therefore, the aim of the present study was to evaluate the ClaID PCR system among clinical *C. auris* isolates recovered in İstanbul, Türkiye, and to investigate the distribution of major *C. auris* clades within this isolate collection. In addition, the study provides one of the first applications of the ClaID framework to a Turkish clinical isolate set and contributes new molecular epidemiological data concerning the circulation of *C. auris* lineages in the region.

## 2. Materials and Methods

### 2.1. Clinical Isolates

A total of 44 non-duplicate clinical *Candida auris* isolates recovered from patients admitted to a tertiary-care hospital in İstanbul, Türkiye, were included in this study. The isolates originated from routine clinical specimens collected during the course of standard patient care and were obtained from the culture collection established in our previous epidemiological investigation of *C. auris* in Türkiye [2]. Initial species identification was performed using matrix-assisted laser desorption/ionization time-of-flight mass spectrometry (MALDI-TOF MS), and all isolates had previously been confirmed by internal transcribed spacer (ITS) region sequencing.

Following identification, isolates were stored at −80 °C in cryoprotective medium until further analysis. Prior to molecular testing, isolates were subcultured on Sabouraud dextrose agar (SDA; Oxoid, Thermo Fisher Scientific, Loughborough, UK) and incubated at 37 °C for 24–48 h to obtain fresh colonies for DNA extraction and PCR analysis.

### 2.2. Genomic DNA Extraction

Genomic DNA was extracted from freshly grown yeast colonies using the High Pure PCR Template Preparation Kit (Roche Diagnostics GmbH, Mannheim, Germany; Cat. No. 11796828001) according to the manufacturer’s instructions. Briefly, a loopful of yeast biomass was transferred into sterile microcentrifuge tubes containing lysis buffer and processed according to the recommended protocol for fungal DNA purification. Following cell lysis, protein digestion, and purification steps, genomic DNA was eluted in nuclease-free water.

The concentration and purity of extracted DNA were evaluated using a NanoDrop spectrophotometer (Thermo Fisher Scientific, Wilmington, DE, USA). DNA preparations were stored at −20 °C until PCR amplification.

DNA quantity and purity were assessed before PCR amplification using spectrophotometric measurements. DNA purity was evaluated based on the A260/A280 ratio, and DNA concentration was recorded as ng/µL. The A260/A280 ratios of the DNA extracts ranged from 1.70 to 1.90, while DNA concentrations ranged from 15.12 to 38.46 ng/µL. These measurements were used to evaluate whether amplification failure in AUS-negative isolates could be associated with low DNA quantity or poor DNA purity.

### 2.3. ClaID PCR Assays

Species confirmation and clade-associated molecular profiling were performed using the ClaID (Clade Identification) PCR framework originally developed by Narayanan et al. [16] and subsequently expanded by the addition of the CSS5 assay for identification of Clade V isolates [17].

The ClaID system consists of a species-specific assay targeting *Candida auris* (AUS-PCR) and clade-specific sequence assays designated CSS1, CSS2, CSS3, CSS4, and CSS5. AUS-PCR was used for molecular confirmation of *C. auris*, whereas CSS assays were designed to identify clade-associated genomic signatures corresponding to Clades I–V.

Primer sequences used in this study are presented in Table 1. Primers for AUS and CSS1–CSS4 assays were adopted from Narayanan et al. [16]. CSS5 primers were adopted from Narayanan et al. [17].

For quality control of the ClaID PCR assays, clade-specific positive-control DNA was included where applicable. Previously characterized reference *C. auris* isolates/DNA representing Clades I–V were used as positive controls for the corresponding CSS assays. The Clade I and Clade V controls included previously published and/or sequence-confirmed isolates, including TMML112 for Clade I and TMML616/TMML617 for Clade V. Clade II, Clade III, and Clade IV assays were controlled using previously characterized clade-confirmed reference material available in our laboratory. No-template controls were included in each PCR run to monitor contamination. The performance and reliability of the PCR assays were evaluated using the corre-sponding positive and negative controls included in each run.

### 2.4. Polymerase Chain Reaction (PCR)

PCR reactions were performed in a final volume of 25 μL containing 12.5 μL of FIREPol^®^ Master Mix Ready to Load (Solis BioDyne, Tartu, Estonia; Cat. No. 04-12-00115), 0.5 μM of each primer, approximately 20–50 ng of template DNA, and nuclease-free water.

Amplifications were carried out using a T100^TM^ Thermal Cycler (Bio-Rad Laboratories, Hercules, CA, USA; Cat. No. 1861096). Cycling conditions were applied according to the original ClaID protocol described by Narayanan et al. [23]. Briefly, reactions consisted of an initial denaturation step at 95 °C for 5 min, followed by 35 amplification cycles comprising denaturation at 95 °C for 30 s, primer annealing at the assay-specific temperature for 30 s, and extension at 72 °C for 30 s. A final extension step was performed at 72 °C for 5 min.

Positive and negative controls were included in each PCR run. Negative controls contained nuclease-free water instead of DNA template to monitor potential contamination.

### 2.5. Agarose Gel Electrophoresis

PCR amplicons were separated by electrophoresis on 1.5% (*w*/*v*) agarose gels prepared in 1× Tris–borate–EDTA (TBE) buffer and stained with a nucleic acid fluorescent dye. Electrophoresis was performed at 100 V for approximately 45–60 min.

A 100 bp DNA ladder (Thermo Fisher Scientific, Waltham, MA, USA) was included in each gel to estimate amplicon sizes. Following electrophoresis, gels were visualized under ultraviolet illumination and photographed using a gel documentation system (Vilber Lourmat, Marne-la-Vallée, France).

Amplification results were interpreted according to the presence or absence of PCR products corresponding to the expected amplicon sizes of the AUS, CSS1, CSS2, CSS3, CSS4, and CSS5 assays as previously described [16,17].

### 2.6. Interpretation of ClaID PCR Results

Isolates yielding the expected AUS-PCR amplification product were considered molecularly compatible with *Candida auris*. Subsequent clade-associated molecular profiling was performed according to the amplification patterns obtained with the CSS1–CSS5 assays.

A positive amplification result with a specific CSS assay was interpreted as the presence of a clade-associated molecular profile corresponding to the respective *C. auris* clade. Thus, amplification with CSS1, CSS2, CSS3, CSS4, and CSS5 was interpreted as molecular profiles compatible with Clades I, II, III, IV, and V, respectively [16,17].

Isolates that produced the expected AUS-PCR product but failed to amplify with any of the clade-specific assays were classified as unassigned by the ClaID framework. Because clade assignment was based on PCR detection of clade-associated genomic markers rather than whole-genome sequencing, results were interpreted as molecular profiles compatible with the corresponding clades.

### 2.7. Statistical Analysis

Descriptive statistical analyses were performed using IBM SPSS Statistics version 26.0 (IBM Corp., Armonk, NY, USA). Categorical variables were summarized as frequencies and percentages.

The proportions of AUS-PCR-positive isolates and the frequencies of clade-associated molecular profiles identified by the CSS1–CSS5 assays were calculated for the entire isolate collection. Results were presented descriptively using counts and percentages.

As the primary objective of the study was the molecular epidemiological characterization of *Candida auris* isolates rather than hypothesis testing or comparison between independent groups, no inferential statistical analyses were performed.

### 2.8. Ethical Considerations

The *Candida auris* isolates analyzed in the present study originated from a previously established and anonymized clinical isolate collection that had been obtained during routine diagnostic procedures. No additional patient recruitment was performed, and no patient-identifiable information was available to the investigators at any stage of the study.

The original collection, identification, and characterization of these clinical isolates were conducted under institutional ethical approval obtained from the Scientific Research Ethics Committee of the University of Health Sciences Ümraniye Training and Research Hospital (Approval No: B.10.1.TKH.4.34.H.GP.0.01/97).

The current study involved only molecular analysis of anonymized fungal isolates and did not include access to clinical records, personal data, or any intervention involving human participants. Therefore, all procedures were conducted in accordance with the principles of the Declaration of Helsinki and relevant institutional regulations governing biomedical research.

## 3. Results

### 3.1. Detection of Candida auris by AUS-PCR

The 44 clinical *Candida auris* isolates analyzed in the present study were recovered from a tertiary-care hospital in İstanbul, Türkiye, during the surveillance period covered by the preceding clinical investigation. The isolate collection represented a regional, single-center dataset and was not intended to reflect the national molecular epidemiology of *C. auris* in Türkiye. Available clinical metadata were reviewed to contextualize the molecular findings, including the clinical origin of the isolates, specimen sources, and the healthcare setting in which the isolates were recovered. Because the present work focused on molecular ClaID-PCR profiling of previously characterized isolates, detailed patient-level outcome analysis and formal outbreak reconstruction were beyond the scope of this study.

A total of 44 clinical *Candida auris* isolates previously identified by MALDI-TOF MS and confirmed by ITS sequencing were evaluated using the AUS-PCR assay. Before PCR amplification, DNA quantity and purity were evaluated for all analyzed isolates. The A260/A280 ratios ranged from 1.70 to 1.90, and DNA concentrations ranged from 15.12 to 38.46 ng/µL. The AUS-negative isolates did not show markedly lower DNA purity or concentration compared with the overall isolate set, with A260/A280 ratios ranging from 1.71 to 1.74 and DNA concentrations ranging from 15.12 to 20.63 ng/µL. Therefore, AUS amplification failure was not clearly associated with insufficient DNA quantity or poor spectrophotometric purity. Of these, 41 isolates (93.2%) produced the expected species-specific amplification product, whereas 3 isolates (6.8%) remained negative despite repeated amplification attempts.

Clade-specific PCR assays were then evaluated using the CSS1–CSS5 primer sets. Among the 44 isolates, 39 isolates (88.6%) generated the expected CSS1 amplification product, indicating an AUS-positive/CSS1-positive Clade I-associated molecular profile. Five isolates did not generate CSS1 amplification despite repeated testing. Based on the combined AUS and CSS amplification profiles, these isolates were interpreted as discordant or unassigned ClaID profiles rather than definitive non-Clade I isolates. No amplification was observed with CSS2, CSS3, CSS4, or CSS5 assays among the clinical isolates tested.

**Figure 1:**
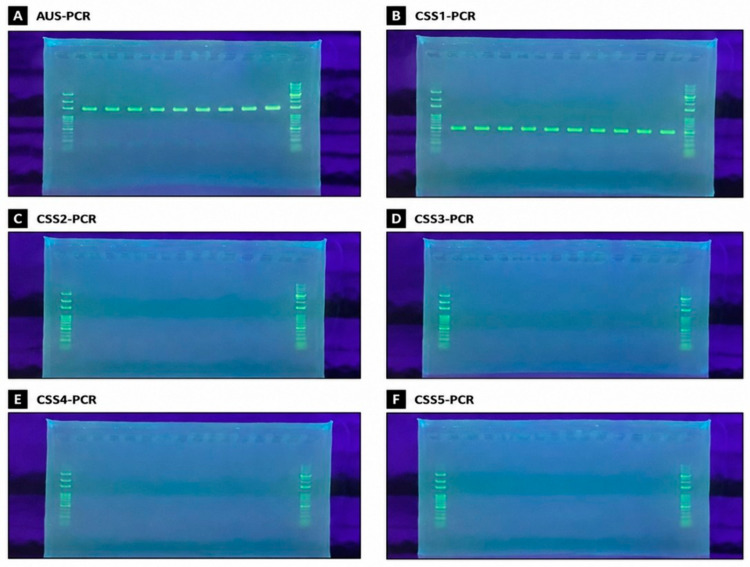
Representative original agarose gel electrophoresis images obtained using AUS and clade-specific PCR assays in clinical Candida auris isolates recovered in İstanbul, Türkiye. (**A**) AUS-PCR. (**B**) CSS1-PCR. (**C**) CSS2-PCR. (**D**) CSS3-PCR. (**E**) CSS4-PCR. (**F**) CSS5-PCR. Amplification products were detected with AUS-PCR and CSS1-PCR assays, whereas no amplification was observed with CSS2, CSS3, CSS4, or CSS5 assays among the analyzed clinical isolates. Lanes: M, 100 bp DNA ladder (left and right in each gel); remaining lanes, clinical isolates; NC, negative control (no template DNA).

**Figure 2:**
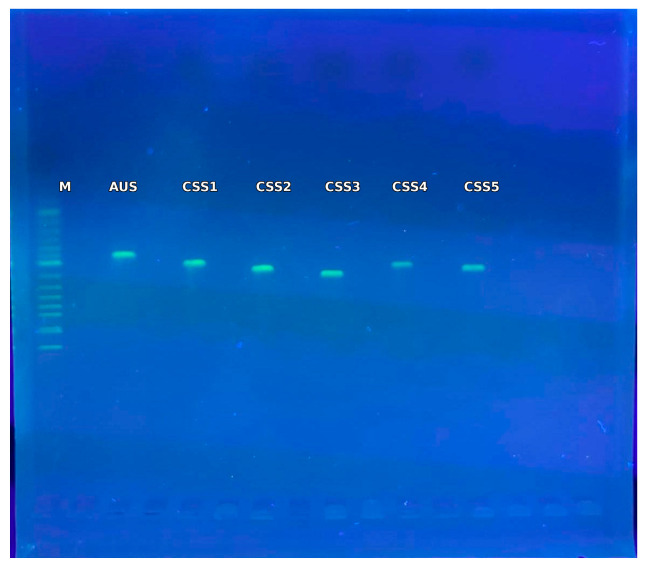
Positive-control amplification patterns for the species-specific AUS assay and clade-specific CSS1–CSS5 PCR assays. Previously characterized clade-confirmed reference material was used as positive-control DNA for the corresponding assays. Lane M: 100 bp DNA ladder; lane 1: AUS positive control; lane 2: CSS1 positive control; lane 3: CSS2 positive control; lane 4: CSS3 positive control; lane 5: CSS4 positive control; lane 6: CSS5 positive control. The expected amplification bands were observed for all positive controls.

Representative AUS-PCR amplification profiles obtained from clinical isolates are shown in Figure 1A. Representative positive-control amplification patterns for the species-specific AUS assay and the clade-specific CSS1–CSS5 assays are presented in Figure 2, confirming the expected performance of the assay controls.

Overall, AUS-PCR results were concordant with the conventional identification results for the majority of isolates included in the study. However, a small subset of isolates failed to produce the expected amplification signal despite prior confirmation as C. auris by reference identification methods.

### 3.2. Clade-Associated Molecular Profiling by ClaID PCR

Clade-associated PCR analysis demonstrated that 39 of the 44 clinical *Candida auris* isolates (88.6%) produced the expected CSS1 amplification product and therefore exhibited a molecular profile compatible with Clade I. Five isolates (11.4%; isolates 11, 13, 23, 44, and M-152) did not yield a detectable CSS1 amplification product despite repeated testing.

No amplification products were detected using the CSS2, CSS3, CSS4, or CSS5 primer sets. Accordingly, no molecular profiles compatible with Clades II, III, IV, or V were identified among the investigated clinical isolates.

Representative amplification profiles obtained using the CSS1 assay are presented in Figure 1B, whereas representative results for the CSS2–CSS5 assays are shown in Figure 1C–F. Representative positive-control amplification patterns for the species-specific AUS assay and the clade-specific CSS1–CSS5 assays are presented in Figure 2, confirming the expected performance of the assay controls.

A summary of the amplification results obtained with the ClaID PCR framework is provided in Table 2.

### 3.3. Distribution of ClaID Molecular Profiles

Evaluation of the combined AUS-PCR and clade-specific PCR results revealed that the predominant molecular profile among the investigated isolates was AUS-positive/CSS1-positive. A total of 39 isolates (88.6%) exhibited this amplification pattern, corresponding to a molecular profile compatible with Clade I.

Two isolates (4.5%) were positive by AUS-PCR but failed to produce amplification with any of the clade-specific assays, including CSS1–CSS5, and were therefore classified as unassigned by the ClaID framework.

Three isolates (6.8%) did not yield the expected AUS-PCR amplification product and consequently could not be further evaluated for clade-associated molecular profiling.

**Figure 3:**
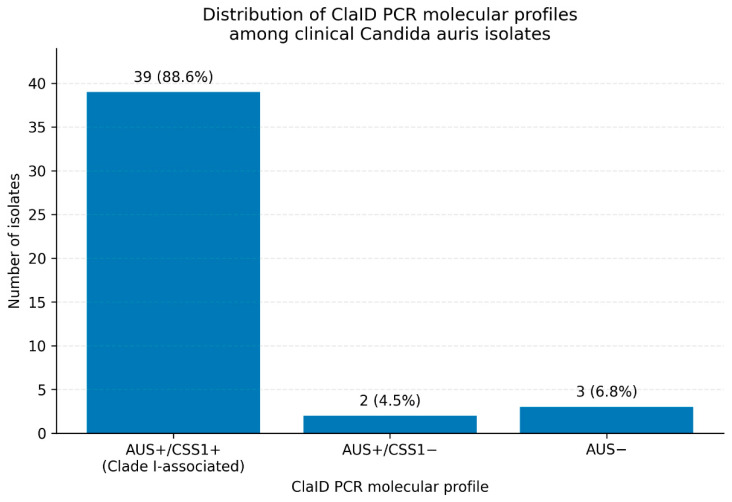
Distribution of ClaID PCR molecular profiles among 44 clinical *Candida auris* isolates recovered in İstanbul, Türkiye. Of the 44 isolates analyzed, 39 (88.6%) exhibited AUS-positive/CSS1-positive amplification profiles, two (4.5%) were AUS-positive but CSS1-negative, and three (6.8%) were AUS-negative. No isolate demonstrated amplification patterns associated with Clades II–V.

The distribution of ClaID molecular profiles among the 44 clinical *Candida auris* isolates is presented in Figure 3.

Among the investigated isolates, the AUS-positive/CSS1-positive amplification pattern represented the most frequently observed ClaID molecular profile. No amplification products were detected with the CSS2, CSS3, CSS4, or CSS5 assays in any isolate included in the study.

## 4. Discussion

*Candida auris* has become an important global fungal pathogen because of its multidrug resistance, capacity for healthcare-associated transmission, environmental persistence, and ability to cause invasive infections in vulnerable patient populations [1,6,7,8,13,14] Since its first description in Japan, *C. auris* has spread across multiple continents and has been increasingly reported in intensive care units and other healthcare environments where prolonged hospitalization, invasive devices, broad-spectrum antimicrobial exposure, and underlying comorbidities facilitate persistence and transmission [2,6,13,14,22] In this context, rapid molecular tools that can support species identification and lineage-level epidemiological screening are becoming increasingly important for infection-control programmes and regional surveillance.

Whole-genome sequencing has demonstrated that the global *C. auris* population is structured into genetically distinct clades that are separated by thousands of single nucleotide polymorphisms and show strong phylogeographic patterns [3,9,10,23,24] Clade I, Clade II, Clade III, Clade IV, and the subsequently described Clade V have been associated with different geographic origins, and recent genomic work has also suggested the emergence of an additional lineage, indicating that the known diversity of the species continues to expand [3,17,23,25] Because clades may differ in antifungal resistance, virulence-associated traits, aggregation phenotypes, and outbreak potential, clade-level identification is relevant not only for phylogenetic studies but also for practical epidemiological surveillance [10,11,12,17,23].

In the present study, the ClaID PCR framework was applied to 44 clinical *C. auris* isolates recovered in İstanbul, Türkiye, which had previously been identified by MALDI-TOF MS and ITS sequencing. The AUS-PCR assay generated the expected species-specific amplification product in 41 of 44 isolates, corresponding to a detection rate of 93.2%. This finding supports the applicability of the AUS assay as a rapid molecular screening tool for *C. auris* identification. However, three isolates did not yield the expected AUS amplicon despite their previous identification by independent methods. Notably, these AUS-negative isolates did not exhibit markedly lower DNA concentration or poorer A260/A280 purity ratios than the overall isolate collection, suggesting that amplification failure was not clearly attributable solely to insufficient DNA quantity or poor spectrophotometric purity. Nevertheless, A260/A280 values provide only a limited assessment of DNA suitability for PCR and do not fully reflect DNA integrity, the presence of PCR inhibitors, or target-region degradation.

Similar discordances may occur in PCR-based assays because of sequence variation in primer-binding regions, DNA integrity issues, low template availability, storage-related effects, or technical amplification failure under routine field conditions [4,16,17,26] Therefore, AUS-negative results in isolates previously identified as *C. auris* should be interpreted cautiously and ideally confirmed by repeat extraction, repeat testing, target-region sequencing, whole-genome sequencing, or alternative validated diagnostic approaches. The most important finding of this study was the predominance of CSS1-positive profiles among the İstanbul clinical isolates. Thirty-nine isolates produced the expected CSS1 amplicon, suggesting a molecular profile compatible with Clade I. No amplification was detected with CSS2, CSS3, CSS4, or CSS5 assays, indicating that no Clade II-, Clade III-, Clade IV-, or Clade V-associated molecular signatures were detected within this isolate collection. This distribution is consistent with the global epidemiological importance of Clade I, which has been widely reported in South Asia, the Middle East, Europe, and other regions and is frequently associated with healthcare-associated outbreaks and multidrug-resistant phenotypes [3,10,18,23,27].

The predominance of a Clade I-associated PCR profile in the present collection is compatible with regional data suggesting that *C. auris* cases reported in Türkiye and neighbouring regions frequently involve lineages related to the South Asian clade. Since the first reported cases in Türkiye, an increasing number of clinical isolates have been identified from healthcare settings, particularly in tertiary-care hospitals and intensive care units [11,12,22,28]. Molecular investigations have further demonstrated the presence of Clade I-associated isolates in the country. Using whole-genome sequencing, Erturk Sengel et al. [11] showed that a multidrug-resistant Turkish clinical isolate belonged to Clade I, while Erkose Genc et al. [12] reported that the first *C. auris* isolates identified in Türkiye exhibited phenotypic and antifungal susceptibility characteristics largely compatible with Clade I strains. More recently, Akkaya et al. [2] reported the predominance of Clade I among a larger Turkish clinical isolate collection. The present study expands these observations by providing a focused PCR-based assessment of clade-associated molecular profiles in clinical isolates recovered in İstanbul. However, because the current isolate set was restricted to a single tertiary-care center in İstanbul and clade assignment was based on PCR amplification profiles rather than whole-genome sequencing, these findings should be interpreted as evidence for the predominance of Clade I-associated molecular profiles within this regional collection, rather than as definitive proof of nationwide clade distribution in Türkiye. Broader multicentre studies incorporating isolates from different geographical regions and whole-genome sequencing will be required to define the national molecular epidemiology of *C. auris* more accurately.

Importantly, the isolates analyzed in the present investigation originated from the same anonymized clinical isolate collection that had previously been characterized with respect to patient demographics, specimen types, antifungal susceptibility profiles, and clinical manifestations [2]. Therefore, the present study should be regarded as a molecular epidemiological extension of that previous investigation, providing clade-associated molecular characterization of an already well-defined clinical isolate collection. The integration of microbiological, clinical, antifungal susceptibility, and molecular epidemiological findings provides a comprehensive framework for understanding the emergence and dissemination of *Candida auris* in Türkiye and may facilitate future national surveillance initiatives [2,16,17].

The absence of CSS2, CSS3, CSS4, and CSS5 amplification among the clinical isolates suggests limited clade diversity within this collection. This finding may reflect the circulation of a dominant lineage in the investigated healthcare setting. Similar patterns have been reported in several regions where local *C. auris* epidemiology is dominated by a single major clade, although international movement of patients and healthcare-associated transmission can introduce additional lineages over time [3,9,23,24]. For this reason, continued surveillance using molecular or genomic tools remains important, particularly in large metropolitan centres such as İstanbul, where patient mobility and referral networks may facilitate the introduction of genetically distinct lineages.

Five isolates did not yield CSS1 amplification. Of these, two were AUS-positive but CSS1-negative, whereas three were AUS-negative. The AUS-positive/CSS1-negative pattern may reflect sequence variation within primer-binding regions, amplification failure at the clade-specific target, or the presence of genomic characteristics not fully captured by the current assay design. The original ClaID approach relies on clade-specific genomic regions identified through comparative whole-genome analyses; therefore, rare polymorphisms or structural variation within these targets may influence amplification efficiency [16]. Similarly, the recently developed CSS5 assay was designed to identify Clade V isolates and expand the discriminatory capacity of the ClaID framework [17]. These findings highlight that PCR-based clade assignment represents a valuable rapid-screening approach but should not be considered equivalent to whole-genome sequencing when discordant or unexpected amplification profiles are encountered.

The inclusion of CSS5 in the present study is particularly important because Clade V represents a genetically distinct lineage separated from previously recognised clades by thousands of SNPs [3,17,25]. Although no CSS5-positive isolate was detected in the present collection, incorporation of this assay into the ClaID framework provides a more comprehensive surveillance strategy. This may be particularly relevant for countries located at the intersection of Europe, Asia, and the Middle East, where population mobility and international healthcare-associated transmission may contribute to the introduction of emerging *C. auris* lineages [17,25] The inclusion of positive-control amplification for AUS and CSS1–CSS5 assays, shown in Figure 2, further supports the technical validity of the assay setup, although PCR-based profiling remains insufficient for definitive genomic clade assignment in the absence of WGS.

Compared with whole-genome sequencing, the ClaID PCR approach offers several practical advantages. WGS remains the reference standard for definitive clade assignment and outbreak reconstruction but requires sequencing infrastructure, bioinformatics expertise, greater financial resources, and longer turnaround times [3,16,17] In contrast, PCR-based methods can be implemented in routine diagnostic laboratories and provide rapid, cost-effective, and easily interpretable results for preliminary epidemiological screening [4,16,17,26] This characteristic is particularly valuable in outbreak investigations and in settings where access to genomic technologies remains limited.

Several limitations should be considered when interpreting the present findings. First, the isolates originated from a regional clinical collection from İstanbul and therefore may not fully represent the national diversity of *C. auris* circulating in Türkiye. Second, clade assignment was inferred from PCR amplification profiles rather than independently confirmed by whole-genome sequencing. Therefore, the designation “Clade I-associated molecular profile” is more appropriate than definitive genomic clade assignment. Third, the discordant AUS-negative and CSS1-negative isolates were not subjected to genomic confirmation, and the molecular basis of these discordant amplification patterns remains unclear. Finally, although the isolates originated from a previously characterized clinical collection, the present study did not directly analyse associations between ClaID molecular profiles, antifungal susceptibility, treatment response, mortality, or other clinical outcomes. Future multicentre studies integrating ClaID PCR with WGS-confirmed clade assignment, antifungal susceptibility testing, and detailed clinical outcome data are needed to validate these findings and to clarify the clinical and epidemiological significance of circulating *C. auris* lineages in Türkiye [2,3,16,17].

## 5. Conclusions

In conclusion, this study provides a field evaluation of the ClaID PCR framework among 44 clinical *Candida auris* isolates recovered from a tertiary-care hospital in İstanbul, Türkiye. The majority of isolates exhibited an AUS-positive/CSS1-positive amplification pattern, indicating a predominance of Clade I-associated molecular profiles within this regional isolate collection. No amplification profiles compatible with Clades II, III, IV, or V were detected among the analyzed clinical isolates. These findings support the potential utility of ClaID PCR as a rapid, accessible, and cost-effective tool for preliminary clade-associated molecular screening and local epidemiological surveillance of *C. auris*.

However, because clade assignment in the present study was based on PCR amplification profiles rather than whole-genome sequencing, the results should be interpreted as clade-associated molecular profiles rather than definitive genomic clade designations. The AUS-negative and CSS1-negative isolates further highlight the need for confirmatory approaches when discordant amplification patterns are encountered. Future multicentre studies incorporating larger isolate collections from different geographical regions of Türkiye, whole-genome sequencing, antifungal susceptibility data, and clinical-epidemiological information will be important to confirm circulating lineages and to better define the clinical and infection-control relevance of *C. auris* in the country.

## Figures and Tables

**Table 1:** Primer sequences used in the ClaID PCR framework for molecular identification and clade-associated profiling of Candida auris.

Assay	Primer	Sequence (5′–3′)	Target	Reference
AUS	AUS-FP	AGAGTCGAGTGAGTCAAAAC	*C. auris*	[23]
AUS	AUS-RP	CTCAACTCGGAATTTTTCATC	*C. auris*	[23]
CSS1	CSS1-FP	TTATTTGGTCTTCAATCATTGATTCCTTGC	Clade I	[23]
CSS1	CSS1-RP	TACGTGTAGTGAGTAGGAATTGAGG	Clade I	[23]
CSS2	CSS2-FP	AGCTACACAAAATGGTTTTTTCAGAT	Clade II	[23]
CSS2	CSS2-RP	CACATCATATGCCAAAGTAGTAGAGT	Clade II	[23]
CSS3	CSS3-FP	CGATGAGAAACCCCCATCCAA	Clade III	[23]
CSS3	CSS3-RP	TTTTCATTTCTATCAGTCAATACAATACGACC	Clade III	[23]
CSS4	CSS4-FP	GGGGGTTTTACTATATAAATTTGTATAGCTT	Clade IV	[23]
CSS4	CSS4-RP	CTATGTAGGTCGGGATTTTCATCC	Clade IV	[23]
CSS5	CSS5-FP	CGTTCTGGCTTACACTAGAAC	Clade V	[24]
CSS5	CSS5-RP	GCCAGCACTACTCCTATCATCA	Clade V	[24]

Primers for AUS and CSS1–CSS4 assays were adopted from Narayanan et al. (2022) [16]. CSS5 primers were adopted from Narayanan et al. (2024) [17].

**Table 2:** Summary of ClaID PCR results obtained from 44 clinical *Candida auris* isolates recovered in İstanbul, Türkiye.

Variable	Number (%)
Clinical isolates analysed	44 (100)
AUS positive	41 (93.2)
AUS negative	3 (6.8)
CSS1 positive	39 (88.6)
CSS1 negative	5 (11.4)
CSS2 positive	0 (0.0)
CSS3 positive	0 (0.0)
CSS4 positive	0 (0.0)
CSS5 positive	0 (0.0)

Three isolates (48, 55, and M-144) did not produce the expected AUS-PCR amplicon. Five isolates (13, 23, 11, 44, and M-152) failed to generate a CSS1 amplification product. No isolate yielded amplification with CSS2, CSS3, CSS4, or CSS5 primer sets.

## Data Availability

The data presented in this study are available from the corresponding author upon reasonable request. All relevant data supporting the findings of this study are included within the article.
